# Measuring attentional bias in smokers during and after psychosocial stress induction with a Trier Social Stress Test in virtual reality *via* eye tracking

**DOI:** 10.3389/fpsyg.2023.1129422

**Published:** 2023-03-31

**Authors:** Benedikt Schröder, Andreas Mühlberger

**Affiliations:** Department for Psychology, Clinical Psychology and Psychotherapy, University of Regensburg, Regensburg, Germany

**Keywords:** smoking, addiction, attention bias, stress, Trier Social Stress Test, eye tracking, virtual reality

## Abstract

**Introduction:**

Attentional bias (AB) is considered an important factor not only in the etiology of addiction, but also with respect to relapse. However, evidence for the predictive ability of AB for relapse is not robust. One reason for this might be fluctuations of AB due to stress. Therefore, the current study investigated whether AB was present during and after stress induction and whether AB was enhanced by stress induction.

**Methods:**

A Virtual Reality (VR) adaptation of the Trier Social Stress Test (VR-TSST) was used to induce psychosocial stress in smokers (*n* = 34) and non-smokers (*n* = 37) followed by a novel free-viewing task in VR. Eye tracking data was recorded to examine gaze behavior to smoking-related and neutral stimuli presented in the VR-TSST and the free-viewing task.

**Results:**

Stress ratings increased significantly from baseline to post VR-TSST in smokers and non-smokers. During the VR-TSST we observed, more frequent, longer, and earlier fixations on smoke-related compared with neutral stimuli without significant group differences. However, in the free-viewing task following the stress induction, a specific AB of smokers in terms of earlier and longer fixations on smoke stimuli was found.

**Conclusion:**

Results indicate that AB is not a persistent trait in smokers, but is context dependent. It is suggested that emotional learning processes such as smoking in the context of relief after stress may contribute to changes of AB both in terms of increased initial attention and deeper stimulus processing. Additionally, the potential of the VR-TSST to induce psychosocial stress could be replicated.

## 1. Introduction

Attention bias (AB) is a phenomenon in which substance users perceive substance-related cues in a prioritized manner, which is reflected by a rapid directing of attention towards, and/or a prolonged attention on these cues (Field and Cox, 2008; Field et al., 2016). The incentive-sensitization theory (IST) of addiction (Robinson and Berridge, 1993; Berridge and Robinson, 2016) provides an explanatory framework for the development of AB (Field et al., 2016). According to IST, the higher salience of drugs and other drug-related stimuli originates from repeated previous substance use, causing increased dopamine neurotransmission in reward-related brain regions. As a consequence, substance-related cues obtain strong incentive motivational properties that contribute to continued use or, in the attempt to quit, to relapse (Robinson and Berridge, 1993; Berridge and Robinson, 2016).

Several meta-analyses confirmed the presence of AB among users of numerous substances including alcohol, cannabis, cigarettes, cocaine, heroin, and opioids (Cox et al., 2006; Rooke et al., 2008; Zhang et al., 2018). In addition, AB was observed in behavioral addiction disorders such as internet-gaming disorder (Kim et al., 2019) and internet-pornography-use disorder (Pekal et al., 2018). Although various theoretical models of AB including IST predict that the strength of AB is indicative of the likelihood of relapse, empirical studies show that the ability of AB to predict future substance use or relapse is not robust (Field et al., 2016). It is suggested that this phenomenon may be explained by conceptualizing AB not as a stable trait but as fluctuating with the motivational state of substance users and by considering methodological issues (i.e., insufficient reliability of traditional methods to measure AB). Given that AB may be dependent on situational or motivational factors, congruence of the situations which are linked to continued use or relapse, and the measurement situation in the laboratory is important to enhance ecological validity (Field et al., 2014, 2016). Indeed, the fluctuating nature of AB is supported by a meta-analysis reporting a significant association between AB and subjective craving (Field et al., 2009). Regarding smoking, previous research additionally showed that AB can be increased by nicotine deprivation (Field et al., 2004; Freeman et al., 2012; Hindocha et al., 2018), alcohol intake (Field et al., 2005), smoking cue exposure (Field et al., 2007), and the anticipation of a smoking opportunity (Wertz and Sayette, 2001). Field et al. (2014, p. 228) conclude in their review that “attentional bias may peak during “high-risk” situations for relapse alongside increases in subjective craving.”

Obvious risk situations are situations associated with stress. Stress is known to play a central role in substance abuse and relapse (Sinha, 2001, 2007; for a recent review see Ruisoto and Contador, 2019). Smokers typically report that stress relief is their primary motive for smoking (e.g., McEwen et al., 2008), that they smoke more when feeling stressed (Kassel et al., 2003), and that stress often triggers the habit of smoking (Taylor et al., 2021). In addition, it was also experimentally shown that smokers smoke more intensely as a result of stress (McKee et al., 2011). Accordingly, smokers commonly believe that smoking is able to reduce stress and negative affect, although research provides very contradictory results as to whether this is actually the case (Kassel et al., 2003; Baker et al., 2004; Hajek et al., 2010). The belief in stress reduction through smoking constitutes one of the most important barriers to smoking cessation (Twyman et al., 2014). In addition, it was found that stress increases cigarette craving, and that relapse often occurs as a result of stressful situations (Baker et al., 2004; Schultz et al., 2022). Despite this important influence of stress on smoking, evidence regarding the influence of stress on AB is scarce. To our knowledge, only one study has examined the influence of stress on AB in smokers. McCarthy et al. (2009) measured AB in smokers and non-smokers with an Addiction-Stroop task and conceptualized stress induction as administration of electric shocks. While the authors found no effect of stress on AB, there are some methodological issues that need to be taken into account. First, the Stroop task provides only an indirect measure of AB (see below for a detailed discussion). Second, and more importantly, the experimental stress induction might have been too dissimilar to real stress situations to which smokers are commonly exposed. Thus, in the present study, we aimed to induce psychosocial stress. The Trier Social Stress Test (TSST), originally developed by Kirschbaum et al. (1993), is considered the gold standard in the induction of acute psychosocial stress (Allen et al., 2017). A stress response indicated by increased cortisol levels is caused in particular by the factors uncontrollability and social-evaluative threat (Dickerson and Kemeny, 2004), and these are realized in the TSST by a previously unannounced arithmetic task and a job interview in front of a committee. Despite numerous deviations and adaptations from the original protocol used over time (Goodman et al., 2017; Narvaez Linares et al., 2020), the TSST generates a consistent physiological and psychological stress response (Allen et al., 2017). The TSST has also been successful in inducing psychosocial stress when implemented in virtual reality (VR), thereby allowing for higher experimental control and standardization. Considering the present study, another advantage of VR is that it allows to place relevant stimuli in the virtual environment while simultaneously measuring gaze *via* an eye tracking device integrated into the head-mounted display (HMD). Consequently, one can measure AB during stress induction. In order to additionally enable a pre-post comparison of the AB within the VR-TSST, we adapted the procedure of the TSST by adding an identically designed 1-min waiting period in which smoking-related and neutral stimuli were visible before receiving the instructions and after conducting the job interview.

The direct measurement of AB *via* eye tracking is considered advantageous in terms of reliability and construct validity over the two paradigms most commonly used in previous research on AB (Field et al., 2014; Drobes et al., 2019), namely the aforementioned Addiction-Stroop task (Cox et al., 2006) and the Visual Probe task (originally designed in the research of depression and anxiety disorders by MacLeod et al., 1986). In both tasks, AB is not measured directly but inferred from reaction times to pictures or words (substance related or neutral) presented on a computer screen (for a detailed description of the tasks procedure, see, e.g., Field and Cox, 2008). Due to this indirect measurement, difficulties often arise in the interpretation of results of both tasks because alternative explanations besides a specific AB remain unresolved (Field and Cox, 2008). Direct measurement of overt attention by means of eye tracking eliminates this ambiguity and makes it possible to examine both components of AB (i.e., faster engagement and delayed disengagement of attention) separately. In addition, the Addiction-Stroop task and the Visual Probe task showed low internal reliability (Ataya et al., 2012; van Bockstaele et al., 2014). Results of traditional measures of AB relying on the presentation of two-dimensional images or words in a laboratory setting may also generalize less to real-life situations. Therefore, as in a previous study (Schröder and Mühlberger, 2022), we used VR to present more realistic three-dimensional stimuli in a naturalistic setting to increase ecological validity while maintaining experimental control. Additionally, VR increases the relevance of the stimuli for participants as immersion in VR is usually accompanied by the experience of presence (Diemer et al., 2015).

The goal of the present study was to investigate the influence of psychosocial stress on AB towards smoking-related stimuli among smokers relative to non-smokers using an ecological valid VR paradigm. We implemented an adapted version of the VR-TSST that measured participants’ overt attention *via* eye gaze both during the VR-TSST as well as in a free-viewing task following the stress induction. We hypothesized that smokers relative to non-smokers show an AB towards smoking related stimuli during the VR-TSST and the free-viewing task both in terms of initial and maintained attention, and that this effect is more pronounced in the experimental parts following the psychosocial stress induction. Finally, we aimed to address an issue raised by Yaxley and Zwaan (2005), who investigated whether participants’ prior knowledge of the smoking-related focus of their study had an impact on AB. In fact, they found that non-smokers had the same AB towards smoking-related pictures as smokers when the smoking focus of the study was known, but only smokers exhibited AB when the focus was unknown. Similar results were reported by Yan et al. (2009) and Knight et al. (2016) who demonstrated that instruction about the study focus induced an AB. Yet, existing awareness for the study focus among participants of AB studies is often not taken into account in the study design but is mentioned merely as a limitation (e.g., Bonitz and Gordon, 2008; Eastwood et al., 2010; Huang et al., 2020). Therefore, we designed our study in a way that all participants were uninformed about its smoking focus.

## 2. Materials and methods

### 2.1. Participants

A total of 82 participants were recruited at the University of Regensburg. Exclusion criteria were age below 18 or above 40 years, pregnancy, neurological disorders, current or former affective or psychotic disorder, psychopharmacological medication, and dependence of substances other than nicotine. All participants had normal or corrected-to-normal vision (as assessed *via* a vision test in VR). Following these criteria, five participants were excluded as there were indications for a mental disorder in the diagnostic brief interview Mini-DIPS Open Access (Margraf and Cwik, 2017). Due to technical problems that led to no eye tracking data being recorded, two further participants had to be excluded. One participant asked to abort the experimental session because the job interview appeared too frightening to her after receiving the instructions and was subsequently excluded, as were two participants for whom an operating error led to a shorter duration of the experiment. Finally, one participant originally assigned to the non-smoking group was excluded because the questionnaires indicated repeated smoking during the last 2 days before the experiment and the last smoked cigarette 1 h before the experiment. This resulted in a final sample of 71 participants (40 female) consisting of 34 smokers (15 female) and 37 non-smokers (25 female) between the age of 18 and 37 years (*M* = 22.56, *SD* = 3.67). Psychology students received course credit as compensation. The study was approved by the Ethics Committee of the University of Regensburg (no. 19-1,576-101).

Smokers reported to smoke *M* = 9.03 cigarettes per day (*SD* = 4.71, overall range between 2–20 cigarettes per day with 79% being within a range of 5–15). Non-smokers reported currently not to smoke on a regular basis. Among non-smokers 54% (*n* = 20) reported never having smoked a cigarette in their lifetime, further 97% (*n* = 36) reported never having smoked regularly in the past, and 3% (*n* = 1) reported having regularly smoked in the past, but not currently. Mean time since last cigarette of those who did smoke cigarettes in their lifetime was 22.27 months (SD = 23.98, range = 2 days to 6 years with 88% being within a range of 2 months to 6 years). Note that after completion of the experimental tasks within the non-smoking group one participant reported smoking one cigarette 2 days prior to the study and one other participant reported smoking one cigarette 14 days prior to the study. However, since the participant smoking 2 days prior reported never having smoked regularly and not currently smoking regularly, and the participant smoking 14 days prior reported not currently smoking regularly, we did not exclude the relating datasets. Smokers rated the statement “Smoking helps me get over stressful periods” between 0 (do not agree) and 100 (strongly agree) with *M* = 65.29 (*SD* = 25.01), and the statement “I smoke more during stressful periods” with *M* = 75.59 (*SD* = 23.89). Exhalation carbon monoxide (CO) level among smokers was 5.62 ppm (*SD* = 2.93) and, M = 1.54 ppm (*SD* = 0.90) among non-smokers. In the Fagerström Test for Nicotine Dependence (FTND, Heatherton et al., 1991) smokers scored between 0 and 6 (*M* = 1.82, *SD* = 2.01). Smokers started smoking at ages between 13 and 21 years (*M* = 17.35, *SD* = 2.10) and reported having smoked the last cigarette between 45 min and 24 h prior to experiment start (*M* = 3.64 h, *SD* = 5.43). There were no significant group differences regarding gender, *χ*^2^(1, 71) = 3.96, *p* = 0.058. Regarding age, a *t*-test indicated that the group of smokers (*M* = 23.53, *SD* = 3.39) had a higher mean age compared with the group of non-smokers (*M* = 21.68, *SD* = 3.74), *t*(69) = 2.18, *p* = 0.032. Accordingly, age was entered as a covariate in the analysis of the eye tracking data.

The lack of prior data regarding the influence of psychosocial stress on attentional bias in smokers prevents an empirically based estimate of the effect size to calculate a power analysis. As suggested by Brysbaert (2019), we therefore used a medium effect size according to Cohen (1988) as a useful effect size of interest. We consequently calculated our power analysis based on a Cohen’s f of 0.25. Using G*Power 3.1.9.2 with the input of 0 as correlation between repeated measures led to a minimum required sample size of *N* = 66 to reach power greater than 0.80 (0.808) for the Stimulus × Group interaction regarding eye tracking data. As a precaution to compensate for possible data loss, we recruited 82 participants, of whom 71 ended up being included in the analyses. By inserting 0 as correlation between repeated measures, we addressed an issue raised by Aberson et al. (2020), who argue that G*Power default settings underestimate the required sample size due to an erroneous doubling of the correlation between repeated measures.

### 2.2. Apparatus and stimuli

The VR was generated using the Steam Source engine (Valve Corporation, Bellevue, WA, United States) and was presented with the HTC VIVE head mounted display (HTC Corporation, Taoyuan, Taiwan). The HMD has headphones attached and an eye tracking device (SensoMotoric Instruments, Teltow, Germany) integrated. Eye tracking was continuously recorded binocular at a sampling rate of 250 Hz with a trackable field of view of 110° and a typical spatial accuracy of 0.2° (SensoMotoric Instruments, 2016). CyberSession Research 5.8 (VTplus GmbH, Würzburg, Germany) provided the eye tracking data in logfiles and controlled the VR environment. Participants’ view and audio were duplicated for the investigator on a separate computer screen and speaker, respectively.

**Figure 1:**
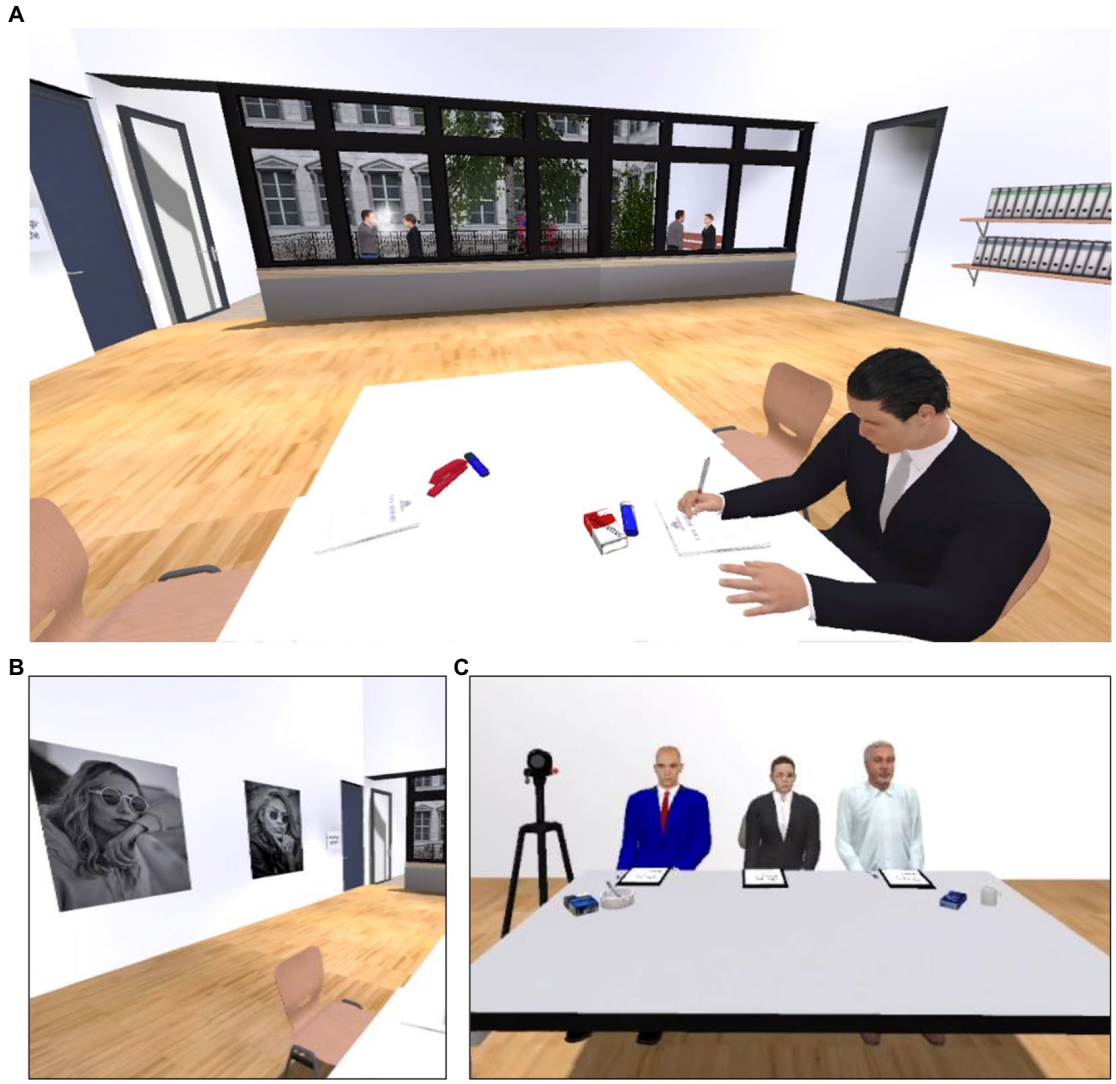
Virtual environment of VR-TSST. **(A)** Preparation room. **(B)** Pictures in the preparation room. **(C)** Job interview committee. Image modified to omit brands.

The VR-TSST consisted of two rooms (see screenshots in Figure 1). In the preparation room, there was a table with chairs at which a competitor was already sitting and taking notes. On one side of the table was a red Marlboro cigarette pack next to a blue lighter. On the opposite side of the table was a red stapler and a blue highlighter. The door to the room where the job interview took place was visible, but yet closed. Next to this door on the wall were two very similar black and white pictures of the same size, each showing a woman wearing sunglasses, one of whom was smoking. Additionally, two female and two male computer characters could be seen through windows with one female talking to each male. One of the two pairs was smoking. In the room of the job interview, a committee consisting of three people (a woman and two men with the woman being in the middle) sat at a table. To the left of the table was a video camera on a tripod. On the table, on one side was a blue Gauloises cigarette pack next to a white ashtray, and on the other side was a blue pack of tissues and a white cup. Thus, there were a total of four smoking-associated regions of interest (ROI) for the analysis of the eye tracking data consisting of ashtray and blue cigarette pack, lighter and red cigarette pack, picture of woman smoking, and window through which the smoking persons were visible. The four corresponding neutral ROIs consisted of cup and tissues, highlighter and stapler, picture of a non-smoking woman, and window through which the non-smoking persons were visible. All stimuli of the ROIs were matched as closely as possible in terms of size, shape, arrangement, and color, and all were counterbalanced in terms of their placement left or right across participants.

In the free-viewing task that followed the VR-TSST, six smoking-associated and six neutral three-dimensional stimuli appeared. The smoke stimuli consisted of an ashtray, a lighter, a Marlboro cigarette pack, a Gauloises cigarette pack, loose cigarettes, and a pack of hand rolling tobacco of the brand Pueblo. The neutral stimuli consisted of a bowl, a highlighter, a stapler, a pack of tissues, pens, and a smartphone.

### 2.3. Procedure

Before arrival, group assignment was performed covertly based on information participants provided in an online prescreening when making an appointment for the experiment. The prescreening contained three questions about smoking status which were embedded alongside five other questions that served as a distraction (alcohol consumption, fear of heights, acting rashly, and two questions about social anxiety). Recruitment took place *via* bulletin boards and social media. In addition, people who smoked were addressed directly on the university campus without disclosing the fact that they were being approached due to their smoking.

Upon arrival at a laboratory of the Department for Psychology at the University of Regensburg, participants were briefed and provided informed consent in written form. Participants were informed that they would be taking part in a virtual job interview, and that the level of stress would be assessed. The actual subject of the study regarding gaze behavior to smoking-related and neutral stimuli was not openly communicated until the debriefing at the end of the experiment (in addition, all questionnaires were administered at the end of the experiment). Likewise, it was not openly communicated until the debriefing that participants were assigned to different groups based on their smoking status. The study took place in a closed laboratory with one investigator per participant who assisted in putting on the HMD, noted the participants’ answers to rating questions, and controlled the VR. After providing informed consent, baseline stress was assessed. Next, the inclusion interview took place. General exclusion and inclusion criteria (neurological disorders, intake of psychopharmacological medication, pregnancy, age between 18 and 40 years) were assessed by the investigators. Additionally, the presence of mental disorders was excluded by conducting the diagnostic brief interview Mini-DIPS Open Access (Margraf and Cwik, 2017). Participants then put on the head mounted display (HMD) and the eye tracker was calibrated. Subsequently, to confirm normal or corrected-to-normal vision, participants were subjected an eye test in which they were asked to read aloud a series of unrelated letters and numbers displayed on the HMD. Participants were then placed in a training room in order to get accustomed to the virtual environment. To reduce the likelihood of cybersickness (Christou and Aristidou, 2017), the participants did not personally navigate, but were teleported to the relevant locations in the VR. Teleporting was first explained and experienced in the training room. Then, participants completed the VR-TSST followed by a free-viewing task.

#### 2.3.1. VR-TSST

Participants started sitting at the table in the VR preparation room and were instructed to wait briefly until they receive further instructions (1 min waiting time). During this time, the participants could look around the preparation room while a competitor, also sitting at the table, made notes on a piece of paper. Afterwards, standardized instructions on the VR-TSST were played over headphones. The procedure of the VR-TSST used in the present study is largely oriented on the standard protocol for conducting the *in vivo* TSST (Kudielka et al., 2007) and is similar to a previous implementation of the VR-TSST (Shiban et al., 2016). Participants were informed that they should imagine an interview for their dream job and should now prepare a 5-min presentation about their own strengths and their particular suitability for the job, which they will later be asked to present to an application committee. The participants were told that they now have a brief moment to prepare for the presentation. This preparation time lasted 3 min. Next, a female character entered the preparation room and announced that the preparation time is over and the presentation is about to begin. As the presentation took place in a standing position, the participants were assisted by the investigator to stand up from their chair. Then, participants were teleported to a position 2 m in front of the committee, from which the participants were asked to start their presentation. If participants did not fully use the 5-min presentation time, they were informed by the committee (triggered by the investigator) that they still had time and were asked to continue their presentation. The presentation was followed by a previously unannounced arithmetic task introduced by the committee, which required to continuously subtract 17 from 2023. The arithmetic task lasted 5 min and participants were instructed to calculate as quickly and correctly as possible and were prompted by the committee to start again at 2023 if they made a mistake. After that, a teleport back to the table of the preparation room took place (participants were assisted by the investigator to sit down) and the participants received the instruction “*Please wait a moment, it will continue shortly*” (again 1 min waiting time). After the waiting period, the free-viewing task was announced as the next task.

#### 2.3.2. Free-viewing task

**Figure 2:**
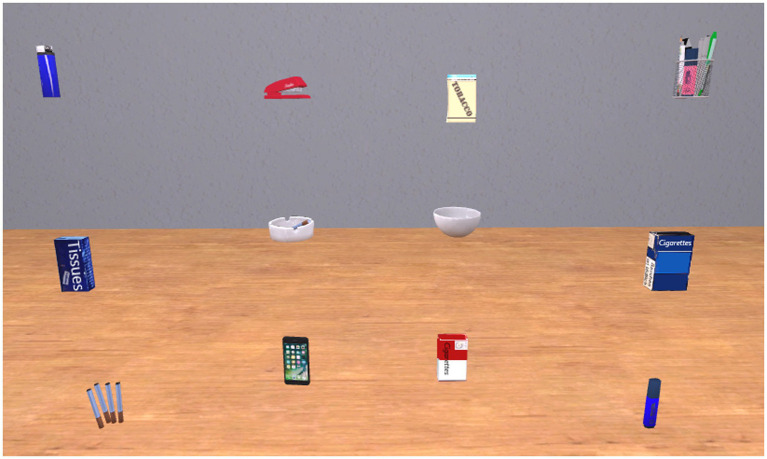
Presentation of smoking-associated and neutral stimuli in the free-viewing task. Image modified to omit brands.

After 1 min of getting accustomed to the new VR environment consisting of a room without furnishings with a wooden floor and gray walls to ensure sufficient contrast also for white stimuli, participants were instructed to look at the objects which were about to appear and to rate their presence between each trial. These ratings aim to mask the intention of the measurement. In each of six trials, a total of 12 objects (6 smoking-related, 6 neutral) were presented simultaneously in a 4 × 3 pattern (see Figure 2) for a duration of 30 s. The same smoking-associated and neutral stimuli as in the VR-TSST part were used (see Stimuli section). To ensure that the outer stimuli were at the same distance from the participants as the inner stimuli, they were placed in a semicircle around the participants’ point of view. Before the start of each trial by the investigator, a blue cube was displayed centrally, which the participants were asked to fixate as a standardized starting point. The locations of the subsequent appearance of the stimuli were pseudorandomized across trials (with, in each row, two smoking-associated and two neutral stimuli; each stimulus appearing only once per position; three smoking-associated and three neutral stimuli in each of the left and right halves of all objects, respectively; in the two central positions, a smoke-associated stimulus with its matched neutral stimulus in the neighboring position) and counterbalanced across participants (all appearance locations of smoking-associated and neutral stimuli were exactly the opposite for one half of the participants as for the other half).

Finally, participants removed the HMD and filled in the questionnaires described below. After completing the experimental procedure, the CO measurement took place, and subsequently, the participants were fully debriefed. The total duration of the experiment was approx. 60 min.

### 2.4. Measures

#### 2.4.1. Questionnaires

Following the free-viewing task, presence was measured with the Igroup Presence Questionnaire (IPQ; Schubert et al., 2001). The occurrence of cybersickness was assessed with the Virtual Reality Sickness Questionnaire (VRSQ; Kim et al., 2018). Additional self-report measures included a demographic questionnaire, a smoking questionnaire documenting smoking history and current smoking behavior, the brief Questionnaire of Smoking Urges (QSU-b; Cox et al., 2001), and the Fagerström Test for Nicotine Dependence (FTND, Heatherton et al., 1991). QSU-b and FTND were administered to smokers only.

#### 2.4.2. Ratings

The perceived stress level was verbally rated five times (baseline before inclusion interview, after receiving VR-TSST instructions, after performing VR-TSST, prior to first trial of free-viewing task, and after completing free-viewing task). At this, participants rated the statement “*On a scale of 0 to 100, how stressed do you feel right now? 0 means not stressed at all and 100 means very stressed*.” One item of the IPQ (Schubert et al., 2001) is used as verbal presence rating between trials of the free-viewing task (“*Between 0 and 100, how strongly do you agree with the following statement: In the computer-generated world, I had the impression of being there. 0 means not at all, and 100 means very strongly*.”). Cigarette craving for the present moment was rated between 0 and 100 after the free-viewing task, and in order not to reveal the relevance of smoking in advance, in retrospect for the beginning of the experiment and for the time directly after the VR-TSST.

#### 2.4.3. Physiological measures

Binocular eye movement data was recorded continuously beginning after a 5-point calibration of the integrated eye tracker once participants put on the HMD. To objectify the smoking status, breath CO measurement (CO Check Pro, MD Diagnostics Ltd., Kent, UK) took place at the end of the experiment for all participants.

### 2.5. Data reduction and statistical analysis

CyberSession Research 5.8 (VTplus GmbH, Würzburg, Germany) provided a single log file for each participant containing eye tracking data (timestamps, gaze angles, ROI in focus, markers of the VR-TSST phases and the stimulus onsets of the free-viewing task). To classify fixations, we used the event detection algorithm REMoDNaV (Dar et al., 2021). This algorithm is an adaptive velocity based algorithm based on an earlier algorithm by Nyström and Holmqvist (2010), which was found to be among the best in an evaluation of several event-detection algorithms (Andersson et al., 2017). Dar et al. (2021) further developed this existing algorithm to be compatible with prolonged recordings of dynamic stimulation. Since this algorithm requires coordinates as input, we converted the gaze angles in our data into coordinates using an in house written script in Python (version 3.8; Python Software Foundation, Delaware, US). The resulting file was analyzed with REMoDNaV and the classified events were then combined with the timestamps, ROI information and markers from the original log files. These combined files were then processed with an additional in house written Python script which determined the number of fixations for each ROI (fixation count), the mean time of one fixation, the time until the first fixation of a ROI (initial fixation time), and the dwell time (i.e., overall time spent on a ROI). This output was subsequently imported in SPSS 26.0 (IBM Corp., Armonk, NY, United States). For the free-viewing task, we additionally determined whether the first fixation of a trial was smoke-related or neutral and calculated the proportion of trials in which participants fixated smoking-related stimuli first. Eye tracking data was subsequently analyzed using 2 × 2 mixed design ANCOVAs (covariate age) with group (smokers, non-smokers) as the between-subjects variable and stimulus (smoking-related, neutral) as the within-subjects variables. ANOVAs were used to examine the main effects of stimulus alone. Potential changes between the initial 1-min waiting period and the 1-min waiting period at the end of the VR-TSST were analyzed with a 2 × 2 × 2 ANCOVA with the additional within-subjects factor time (pre, post). When examining the main effects of the within factors time and stimulus without the factor group, ANOVA results are reported as the covariate age is, at that point, not required. The proportion of smoking-first fixations across trials of the free-viewing task was analyzed by calculating a univariate ANCOVA with the covariate age. For a manipulation check of the stress induction, we calculated a Group × Time ANOVA over the five time points of the stress ratings. Greenhouse–Geisser correction was used if necessary. Partial η^2^ ($\eta_{p}^{2}$) scores are reported as effect sizes. Alpha level was 5% for all statistical analyses. As an increasing number of researchers suggest (e.g., Aczel et al., 2018; Wagenmakers et al., 2018), we calculated Bayes factors (BF_01_) to quantify support of the null-hypotheses (*H_0_*) for non-significant results including age as covariate. Bayesian analysis was conducted in JASP (Version 0.16.4; JASP Team, 2022). Resulting Bayes factors were interpreted according to the categories of Jeffreys (1961).

## 3. Results

### 3.1. Stress, craving and VR characteristics

**Figure 3:**
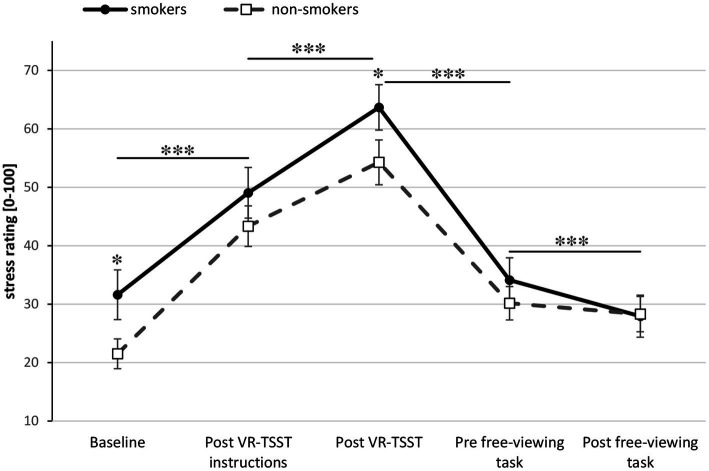
Stress ratings during the course of the experiment for smokers (*n* = 34) and non-smokers (n = 37). Asterisks indicate significant differences. Error bars show standard errors.

Regarding stress ratings (see Figure 3), a Group × Time ANOVA revealed a significant main effect Time [*F*(4, 276) = 63.15, *p* < 0.001, $\eta_{p}^{2}$ = 0.478]. *Post hoc* ANOVAs indicate a significant increase in stress from baseline to post VR-TSST instructions [*F*(1, 69) = 65.39, *p* < 0.001, $\eta_{p}^{2}$ = 0.487], a further significant increase from post VR-TSST instructions to post VR-TSST [*F*(1, 69) = 22.02, *p* < 0.001, $\eta_{p}^{2}$ = 0.242], a significant decrease from post VR-TSST to pre free-viewing task [*F*(1, 69) = 114.74, *p* < 0.001, $\eta_{p}^{2}$ = 0.624], and another significant decrease from pre to post free-viewing task [*F*(1, 69) = 11.45, *p* = 0.001, $\eta_{p}^{2}$ = 0.142]. Exploratory analyses of the different measurement time points with univariate ANCOVAs (covariate age) revealed significantly higher stress levels in smokers compared to non-smokers at the beginning of the experiment [*F*(1, 68) = 4.63, *p* = 0.035, $\eta_{p}^{2}$ = 0.064] as well as immediately after the VR-TSST [*F*(1, 68) = 4.22, *p* = 0.044, $\eta_{p}^{2}$ = 0.058].

**Figure 4:**
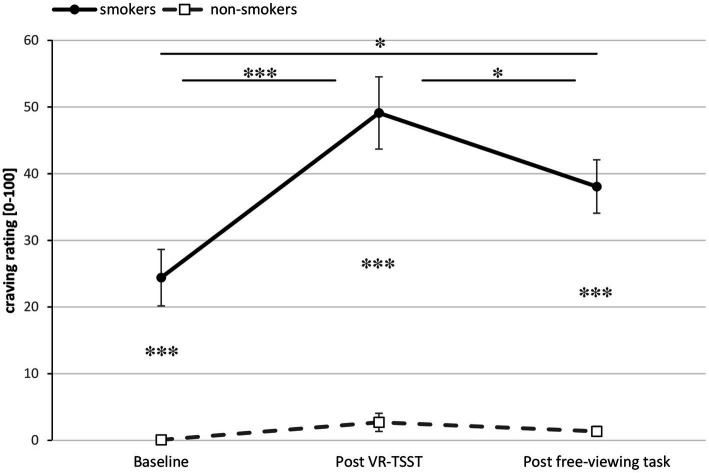
Craving ratings during the course of the experiment for smokers (*n* = 34) and non-smokers (*n* = 37). Ratings at baseline and post VR-TSST were given retrospectively. Asterisks indicate significant differences. Error bars show standard errors.

ANCOVA results for the course of craving yielded a significant Group × Time interaction [*F*(1.49, 101.47) = 7.71, *p* = 0.002, $\eta_{p}^{2}$ = 0.102], indicating significant changes of craving over time for smokers only (see Figure 4). Subsequent *t*-tests showed within smokers a significant increase of craving from baseline to post VR-TSST, *t*(33) = −3.58, *p* < 0.001, a significant decrease from post VR-TSST to post free-viewing task, *t*(33) = 2.64, *p* = 0.012, and a significant increase from pre experiment to post free-viewing task, *t*(33) = −2.69, *p* = 0.011. Additionally, smokers reported significantly higher craving than non-smokers [*F*(1, 69) = 122.63, *p* < 0.001, $\eta_{p}^{2}$ = 0.640]. Exploratively, we examined whether there was an association in smokers between the peak craving and the peak stress, each following the VR-TSST. A Pearson correlation coefficient was computed and yielded a significant positive correlation between the two variables in smokers, *r*(32) = 0.48, *p* = 0.002, but not in non-smokers, *r*(35) = 0.08, *p* = 0.319.

Low levels of VR sickness (the possible maximum score of VRSQ is 27) were reported (*M* = 6.28, SD = 4.26) without significant differences between groups (*p* = 0.647). Medium presence (the feeling of being there; possible IPQ scores ranging from −42 to 42) with M = 1.13 (*SD* = 13.23) was reported without significant group differences (*p* = 0.883).

### 3.2. Gaze during the VR-TSST

Gaze data during the VR-TSST revealed generally more frequent, longer, and earlier fixations on smoke stimuli compared with neutral stimuli (see Table 1 for summary statistics). Accordingly, there were main effects of Stimulus for fixation count [*F*(1, 69) = 15.26, *p* < 0.001, $\eta_{p}^{2}$ = 0.181], mean fixation time [*F*(1, 69) = 14.65, *p* < 0.001, $\eta_{p}^{2}$ = 0.175], initial fixation time [*F*(1, 69) = 4.21, *p* = 0.044, $\eta_{p}^{2}$ = 0.058], and dwell time [*F*(1, 69) = 14.09, *p* < 0.001, $\eta_{p}^{2}$ = 0.170].

**Table 1:** Descriptive results of eye tracking during the VR-TSST.

	Stimuli	Smokers (*n* = 34)		Non-smokers (*n* = 37)	
		*M*	*SD*	*M*	*SD*
Fixation count	Smoke	155.09	104.72	144.89	87.98
	Neutral	91.38	71.21	86.73	55.01
Mean fixation time (ms)	Smoke	152	48	159	57
	Neutral	122	39	124	65
Initial fixation time (s)	Smoke	28.32	18.85	20.78	16.32
	Neutral	32.45	21.27	30.29	19.22
Dwell time (s)	Smoke	83.51	50.90	81.56	47.34
	Neutral	52.16	31.16	53.07	29.60

Means (*M*) and standard deviations (*SD*) as well as number of participants (*n*) are given for smokers and non-smokers. Fixation counts are unitless absolute numbers, milliseconds are indicated for mean fixation time, and seconds are indicated for the remaining time variables.

However, contrary to our expectation, the Group × Stimulus ANCOVAs did not yield significant Group × Stimulus interactions for any of the eye movement variables (fixation count: *p* = 0.846; mean fixation time: *p* = 0.522; initial fixation time: *p* = 0.281; dwell time: *p* = 0.787). The subsequent Bayesian analyses stated moderate evidence for *H_0_* regarding fixation count (BF_01_ = 4.04), mean fixation time (BF_01_ = 3.87) and dwell time (BF_01_ = 4.08), and anecdotal evidence for *H_0_* regarding initial fixation time (BF_01_ = 2.87).

With regard to the comparison of the 1-min waiting time before and after the VR-TSST, the Group × Stimulus × Time ANCOVA for fixation count did not show the expected three-way interaction (*p* = 0.172; BF_01_ = 3.27 indicating moderate evidence for H_0_). Furthermore, there were no significant Group × Stimulus × Time interactions for mean fixation time (*p* = 0.524; BF_01_ = 4.33, indicating moderate evidence for *H_0_*), initial fixation time (*p* = 0.285; BF_10_ = 1.32, indicating anecdotal evidence for *H_1_*), and dwell time (*p* = 0.227; BF_01_ = 3.47, indicating moderate evidence for *H_0_*). Instead, the main effects of Stimulus (more frequent, longer, and earlier fixations on smoke stimuli) were again evident (fixation count: *F*(1, 69) = 13.10, *p* = 0.001, $\eta_{p}^{2}$ = 0.160; mean fixation time: *F*(1, 69) = 24.06, *p* < 0.001, $\eta_{p}^{2}$ = 0.259; initial fixation time: *F*(1, 60) = 10.72, *p* = 0.002, $\eta_{p}^{2}$ = 0.152; dwell time: *F*(1, 69) = 13.63, *p* < 0.001, $\eta_{p}^{2}$ = 0.165). Additionally, there was a main effect Time for mean fixation time [*F*(1, 69) = 6.39, *p* = 0.014, $\eta_{p}^{2}$ = 0.085], indicating a general reduction in mean fixation times from pre to post VR-TSST (pre: *M* = 175 ms, *SD* = 83 ms; post: *M* = 144 ms, *SD* = 77 ms).

### 3.3. Gaze in the free-viewing task

Although smokers showed descriptively more fixations than non-smokers to smoke-related stimuli and at the same time slightly fewer fixations to neutral stimuli (see Table 2), the corresponding ANCOVA for fixation counts yielded no significant Group × Stimulus interaction (*p* = 0.087; BF_01_ = 1.50, indicating anecdotal evidence for *H_0_*). Instead, smoke stimuli were generally fixated more often than neutral stimuli [*F*(1, 69) = 33.81, *p* < 0.001, $\eta_{p}^{2}$ = 0.329].

**Table 2:** Descriptive results of eye tracking during the free-viewing task.

	Stimuli	Smokers (*n* = 34)		Non-smokers (*n* = 37)	
		*M*	*SD*	*M*	*SD*
Fixation count	Smoke	116.21	44.96	108.19	39.04
	Neutral	89.79	35.88	93.00	37.95
Mean fixation time (ms)	Smoke	130	41	113	42
	Neutral	107	34	110	36
Initial fixation time (s)	Smoke	4.36	2.70	5.48	3.10
	Neutral	5.41	2.17	5.31	2.98
Proportion smoke-related first fixation (%)		59.31	17.98	46.40	23.94
Dwell time (s)	Smoke	78.15	12.70	76.43	15.31
	Neutral	67.62	12.16	68.21	12.38

Means (*M*) and standard deviations (*SD*) as well as number of participants (*n*) are given for smokers and non-smokers. Fixation counts are unitless absolute numbers, milliseconds are indicated for mean fixation time, seconds are indicated for the remaining time variables, and percentages are given for the proportion of trials in which first fixations were smoke-related.

**Figure 5:**
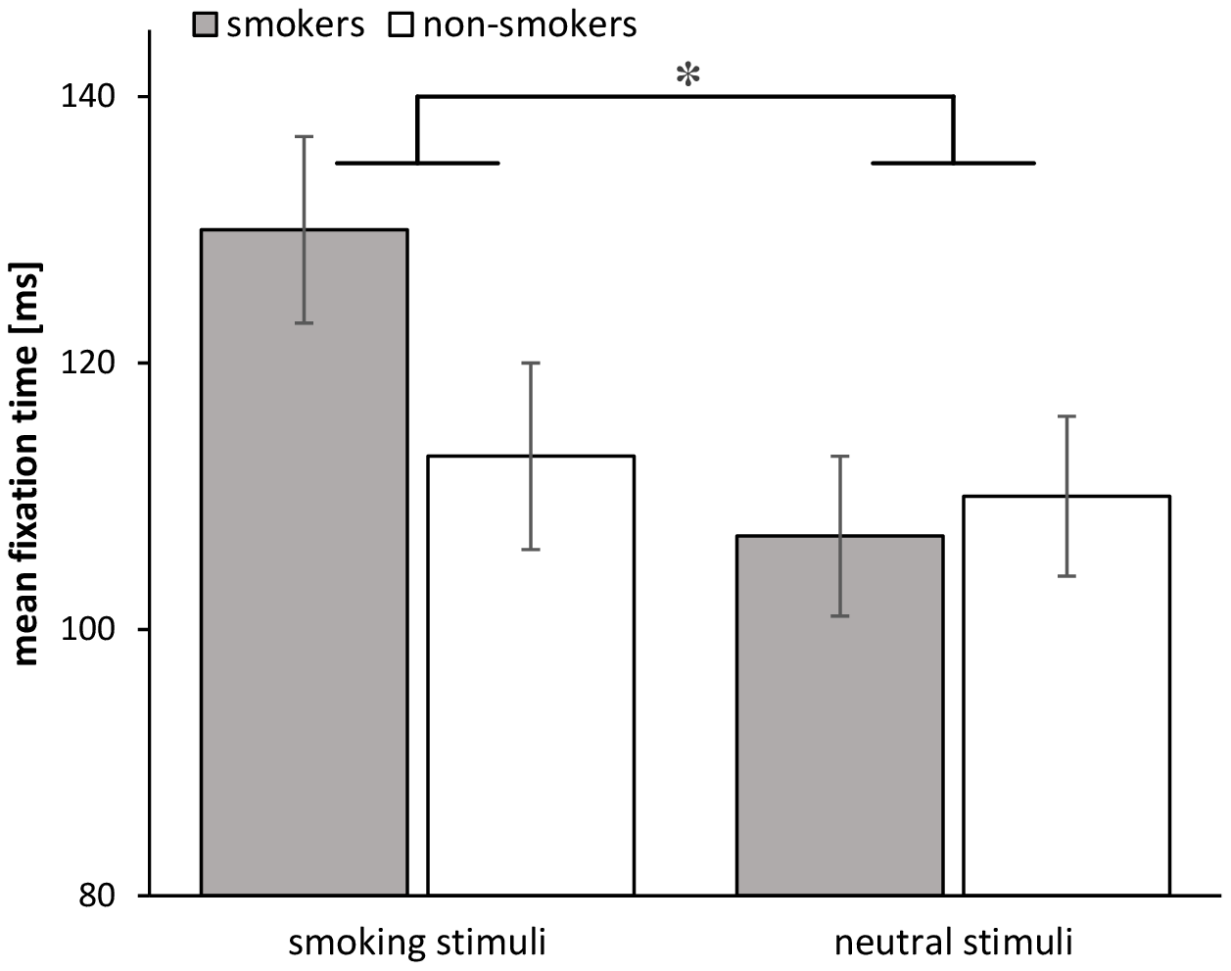
Mean fixation times for smokers (*n* = 34) and non-smokers (*n* = 37) in the free-viewing task. The asterisk indicates the significant interaction. Error bars show standard errors.

There was a significant Group × Stimulus interaction for mean fixation time [*F*(1, 68) = 5.35, *p* = 0.024, $\eta_{p}^{2}$ = 0.073] indicating that the fixation time of smokers to smoke stimuli was higher relative to non-smokers, while the fixation time of non-smokers to neutral stimuli was higher (see Figure 5). Additionally, it showed a significant main effect Stimulus, indicating that mean fixation times of smoke stimuli were higher relative to neutral stimuli [*F*(1, 69) = 12.56, *p* < 0.001, $\eta_{p}^{2}$ = 0.154].

**Figure 6:**
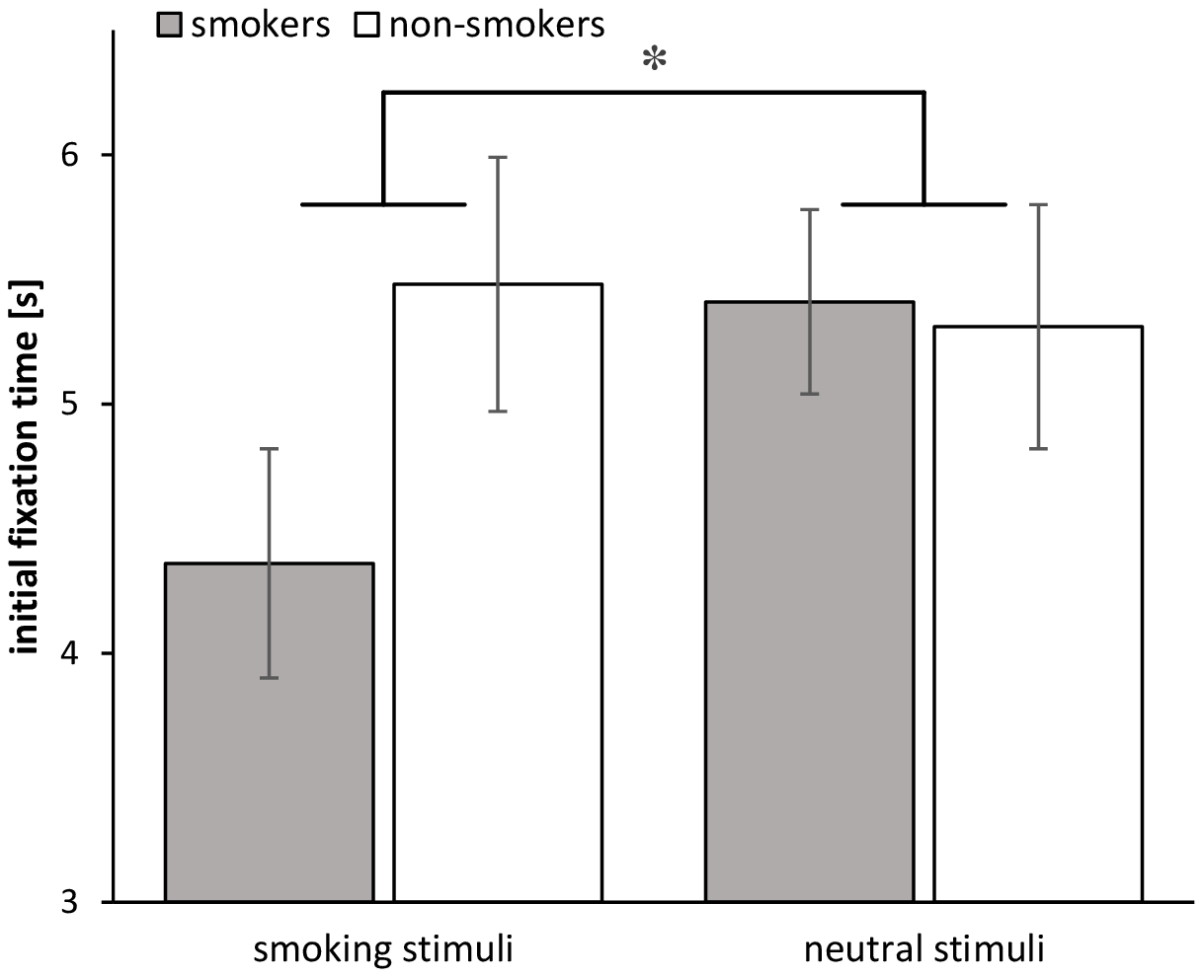
Initial fixation times for smokers (*n* = 34) and non-smokers (*n* = 37) in the free-viewing task. The asterisk indicates the significant interaction. Error bars show standard errors.

Regarding initial fixation time, a further ANCOVA revealed a significant Group × Stimulus interaction [*F*(1, 68) = 5.86, *p* = 0.018, $\eta_{p}^{2}$ = 0.079] consisting of earlier fixations of smokers on smoke stimuli relative to non-smokers, with minimal later fixations of smokers on neutral stimuli (see Figure 6). Additionally, a univariate ANCOVA indicated that the proportion of trials in which participants fixated smoking-related stimuli relative to neutral stimuli first, was significantly higher for smokers than for non-smokers [*F*(1, 68) = 5.62, *p* = 0.021, $\eta_{p}^{2}$ = 0.076].

For dwell time, there was no significant Group × Stimulus interaction (*p* = 0.448; BF_01_ = 3.15, indicating moderate evidence for *H_0_*), but a significant main effect Stimulus [*F*(1, 69) = 50.29, *p* < 0.001, $\eta_{p}^{2}$ = 0.422] indicating generally longer dwell times on smoke stimuli compared with neutral stimuli.

The current results cannot be explained by effects related to a single stimulus but rather reflect effects across all stimuli in each category. The contribution of unique smoke stimuli to total fixations across all participants ranged from 6.15 to 12.82% and the corresponding dwell times ranged from 10.13 to 16.98 s [lighter: fixations: *M* = 6.15% (*SD* = 4.15), dwell time: *M* = 10.13 s (*SD* = 3.22); loose cigarettes: *M* = 6.40% (*SD* = 3.28), *M* = 10.33 s (*SD* = 2.84); ashtray: *M* = 8.64% (*SD* = 4.69), *M* = 11.44 s (*SD* = 3.80); Marlboro cigarette pack: *M* = 9.36% (*SD* = 5.49), *M* = 14.00 s (*SD* = 4.29); hand rolling tobacco pack: *M* = 11.92% (*SD* = 7.00), *M* = 14.37 s (*SD* = 4.23); Gauloises cigarette pack: *M* = 12.82% (*SD* = 5.46), *M* = 16.98 s (*SD* = 4.61)]. The contribution of unique neutral stimuli to total fixations across all participants ranged from 5.14 to 10.38% and the corresponding dwell times ranged from 8.69 to 14.50 s [bowl: fixations: *M* = 5.14% (*SD* = 3.24), dwell time: *M* = 8.69 s (*SD* = 2.83); highlighter: *M* = 5.73% (*SD* = 4.15), *M* = 8.85 s (*SD* = 3.14); stapler: *M* = 5.90% (*SD* = 3.13), *M* = 9.93 s (*SD* = 2.94); pens: *M* = 8.74% (*SD* = 4.28), *M* = 12.66 s (*SD* = 3.44); pack of tissues: *M* = 8.82% (*SD* = 4.31), *M* = 13.30 s (*SD* = 3.79); smartphone: *M* = 10.38% (*SD* = 5.18), *M* = 14.50 s (*SD* = 4.75)].

## 4. Discussion

To the best of our knowledge, the present study was the first to investigate AB in smokers relative to non-smokers during and after a psychosocial stress induction in VR.

In the following we summarize the main findings. With regard to stress ratings, there was a significant increase in stress level for both groups within the VR-TSST, replicating earlier results and indicating successful stress induction. This was followed by a generally significant reduction of stress between the end of the VR-TSST and the start of the free-viewing task. In addition, slightly higher stress levels were reported by smokers relative to non-smokers at baseline as well as after the VR-TSST. Further, smokers reported higher levels of craving than non-smokers. Craving among smokers increased significantly until after the VR-TSST, and decreased significantly until after the free-viewing task. Note, however, that craving was higher at the end than at the beginning of the experiment. Additionally, craving and stress ratings were associated in the smoking group for the time point after completion of the VR-TSST.

Analysis of gaze behavior during the VR-TSST showed more frequent, longer, and earlier fixations on smoking-related stimuli relative to neutral stimuli for all participants (smokers and non-smokers). Thus, during psychosocial stress induction no specific AB was observed exclusively from smokers to smoking-related stimuli, leading us to reject the corresponding hypothesis. Regarding the waiting periods inserted in the VR-TSST before and after stress induction, similar results were observed in terms of generally more frequent, longer, as well as earlier fixations on smoking-related stimuli. The only significant change from pre to post VR-TSST waiting times consisted of a reduction in mean fixation times independent of stimulus class or group. Hence, the eye tracking data of the waiting periods did not show a specific AB, and contrary to our hypothesis, also no increase in AB in the waiting period after the job interview. However, in the free-viewing task applied after the VR-TSST, a specific AB was found with respect to fixation duration, initial fixation time, and the proportion of trials in which smoke-related stimuli were fixated first. No group differences, however, were found with regard to the total duration of fixations (i.e., dwell time), instead smoke stimuli were generally fixated longer than neutral stimuli. There was also no significant difference in number of fixations, although the descriptive results pointed in the direction of an AB in smokers.

Interpreting our results, smoking-related stimuli were generally more salient compared to neutral stimuli to all participants during VR-TSST. This can probably be explained by specific stimulus characteristics. Smoking stimuli might seem more interesting, their presence might be more surprising, and/or they might generally have higher emotional valence. This idea is supported by previous research by Nummenmaa et al. (2006), who showed that participants were more likely to view emotional than neutral images, even when instructed to view the neutral images. An additional factor that may have contributed to the observed main effect of stimulus could be that the presentation of multiple smoking stimuli increased participants’ awareness of the smoking focus of the study and resulted in an AB in non-smokers, even though participants were not given explicit information about the focus of the study (Yaxley and Zwaan, 2005; Yan et al., 2009). Interpreting the non-significant Group × Stimulus interaction as the absence of AB during the VR-TSST involves the possibility of a type II error. However, Bayesian analyses provide some evidence for *H_0_*. If the specific AB is indeed absent here, a possible explanation might be that performance situations like these may not be associated with smoking *during* the performance, as smokers would possibly not smoke while preparing a presentation, and would almost certainly not smoke during the job interview. Thus, the absence of this association would not trigger any substance-seeking behavior in smokers that goes beyond the normal attention of non-smokers. This possibility could also explain why no AB appeared at the time of the waiting period after the job interview (please note again the possibility of a type II error). During this waiting period, the VR-TSST was not yet declared over for the participants (i.e., participants likely maintained focus on the task ahead and still felt pressure to perform), but the instruction was to wait for a short time and that the experiment would be continued in a moment (including the possibility of being immediately contacted again, which is unlikely to be a smoking-associated situation). The overall lower mean fixation time in the waiting period after stress induction is probably due to familiarity with the situation and stimuli, resulting in a less intensive visual inspection after recognition.

Interestingly, in contrast to during the VR-TSST, smokers showed a specific AB to smoking-related stimuli in the free-viewing task after completion of the VR-TSST. AB was evident within the free-viewing task in terms of faster engagement and less consistently in terms of maintained attention. The faster engagement component is reflected by shorter initial fixation times and a higher proportion of smoking-first fixations among smokers (note however, that due to the lack of a trial structure, we cannot compare the free-viewing task to the VR-TSST). Maintained attention is partially reflected in our data by longer mean fixation times of smokers to smoking-related stimuli relative to non-smokers and neutral stimuli. This may imply increased attention of smokers to smoke-related stimuli due to the relevance of the stimuli. However, for other measures of maintained attention (i.e., dwell time and fixation count), our data do not provide evidence for sustained attention regarding smoking stimuli. This suggests that AB may be more pronounced in smokers with respect to initial attention than to maintained attention or that initial attention may be more affected by stress. Two previous studies using visual probe tasks to investigate AB in smokers found biases in initial as well as maintained attention (Bradley et al., 2004; Field et al., 2004), whereas Mogg et al. (2003) found a significant AB only regarding maintained attention. As visual probe tasks are not optimal to differentiate between initial and maintained attention (Field and Cox, 2008), further research using eye tracking as a direct measure is needed to clarify these inconclusive results.

Importantly, the fact that within the same participants initially no AB was observed during the first part of the experiment, but was evident when stress induction was finished, basically supports the notion that AB is not stable but variable. Different explanations can account for this variability within our study. One consideration consists of methodological differences between the VR-TSST and the free-viewing task. Whereas stimuli in the free-viewing task are presented in a mixed way, they are presented clearly separated (e.g., on different sides of a table) in the VR-TSST. If this difference was responsible for the occurrence of AB only in the free-viewing task, this would imply that AB only occurs when smoke stimuli must be detected among several other stimuli. However, this explanation does seem unlikely as AB has so far been shown mostly within visual probe and Stroop tasks (Field et al., 2014), i.e., paradigms presenting stimuli separately.

In contrast, it seems more plausible that the most obvious differences, namely that the VR-TSST but not the free-viewing task induced psychosocial stress and that the free-viewing task was conducted after experiencing psychosocial stress, was related to the observed outcomes. Experiencing psychosocial stress does not seem to directly lead to the amplification of AB as stress levels (and also craving) were already high in smokers within the VR-TSST. Importantly, stress levels had already decreased significantly before the free-viewing task. Thus, we suggest that the mechanism behind the AB in our study is related to the experience of stress relief. Stress relief may act as an internal stimulus for substance seeking behavior as a result of conditioning. Smokers are likely to have previous experiences of smoking after completing a stressful task and the relief that comes with it. Indeed, clinical observations of smoking cessation courses at our department anecdotally point to this association, insofar as smokers regularly report smoking automatically or deliberately as a reward after completing a task. This observation is in line with Fagerström (2012), who argues that the rewarding property of cigarettes after completion of a task is an important function of smoking. The association of finishing a task and smoking is further supported by a study among college students, which reports that taking off one’s mind and taking a break after a stressful event as well as reward after an exam or study session were essential functions of smoking (Nichter et al., 2007).

Successful induction of psychosocial stress is in line with previous studies (e.g., Kotlyar et al., 2008; Shiban et al., 2016; Zimmer et al., 2019) and further highlights the usefulness of the VR-TSST. The fact that smokers reported higher stress levels than non-smokers immediately after the VR-TSST, however, cannot be interpreted as a direct effect of the VR-TSST because stress levels were already elevated among smokers compared with non-smokers at baseline. This finding further supports the existing evidence that active smokers generally report more stress than non-smokers (Hajek et al., 2010).

An important limitation of our study is that its design does not include a no-stress control group. Thus, the influence of stress relief on AB can only be seen as an indication, but not as a proof. Future research could aim to clarify this influence by replicating our approach while additionally introducing a placebo (Het et al., 2009) or friendly TSST version (Wiemers et al., 2013). To further clarify the influence of stress on AB, follow-up work could include the free-viewing task before and after stress induction. This would also enable a comparison of the proportion of smoking-first fixations. Another limitation of our study is that the group of smokers consists of relatively slight smokers (previous studies define this as less than 20 cigarettes daily, which corresponds to our sample; Hogarth et al., 2003; Vollstädt-Klein et al., 2011). It is conceivable that a sample of more severe smokers would have already shown AB during the VR-TSST, which would be consistent with an IST (Robinson and Berridge, 1993) prediction that heavier substance use should be associated with heavier AB. However, this prediction in the context of smoking has not been conclusively clarified as some studies found positive associations between the severity of smoking and the strength of AB, but others found negative or no associations (see review by Field and Cox, 2008). In this regard, a replication of our approach with more severe smokers would be beneficial. Finally, there is a limitation regarding retrospective craving ratings. Retrospectively collected data often have low accuracy and are subject to systematic bias (Ebner-Priemer and Trull, 2009). In order not to disclose the topic of the study in advance, this was, from our point of view, the only possibility to obtain an impression of the craving process nevertheless. Yet, due to the uncertainty involved in retrospective measures, we refrained from putting too much emphasis on craving in our interpretation. Despite the lack of prior information regarding the study’s focus on smoking, we cannot completely exclude awareness of this focus, as it might have arisen due to the large number of smoking-related stimuli. It is important to note, that even though such an effect may explain general effects of AB, we still observed a group-specific AB in the free-viewing task, suggesting differential attentional processing in substance users.

In conclusion, our results indicate that AB is not a persistent trait in smokers, but is context dependent. We suggest that particularly relief after stress promotes substance seeking in smokers and thus contributes to changes of AB both in terms of increased initial attention and deeper stimulus processing. Additionally, the free-viewing task in VR proved valuable as a novel paradigm for measuring AB and the VR-TSST confirmed its previously demonstrated abilities to induce psychosocial stress.

## Data availability statement

The raw data supporting the conclusions of this article will be made available by the authors, without undue reservation.

## Ethics statement

The studies involving human participants were reviewed and approved by Ethikkommission bei der Universität Regensburg. The patients/participants provided their written informed consent to participate in this study.

## Author contributions

BS conceived the presented study idea and was responsible for data acquisition, conducted the data preprocessing and statistical analyses under consultation of AM and wrote the first draft of the manuscript, which was finalized with the contribution of AM. BS and AM developed the study design. All authors contributed to the article and approved the submitted version.

## Conflict of interest

AM is a stakeholder of VTplus GmbH, Würzburg, Germany, that develops and sells virtual environment research systems.

The remaining author declares that the research was conducted in the absence of any commercial or financial relationships that could be construed as a potential conflict of interest.

## Publisher’s note

All claims expressed in this article are solely those of the authors and do not necessarily represent those of their affiliated organizations, or those of the publisher, the editors and the reviewers. Any product that may be evaluated in this article, or claim that may be made by its manufacturer, is not guaranteed or endorsed by the publisher.

## References

[ref1] Aberson C. L. Bostyn D. H. Carpenter T. Conrique B. G. Giner-Sorolla R. Lewis N. A. Jr.. (2020). Techniques and solutions for sample size determination in psychology: supplementary material for “power to detect what? Considerations for planning and evaluating sample size”. Available at: https://osf.io/9bt5s/ (Accessed March 17, 2023). 10.1177/10888683241228328 PMC11193916 38345247

[ref2] Aczel B. Palfi B. Szollosi A. Kovacs M. Szaszi B. Szecsi P.. (2018). Quantifying support for the null hypothesis in psychology: an empirical investigation. Adv. Methods Pract. Psychol. Sci. 1, 357–366. doi: 10.1177/2515245918773742

[ref3] Allen A. P. Kennedy P. J. Dockray S. Cryan J. F. Dinan T. G. Clarke G. (2017). The Trier social stress test: principles and practice. Neurobiol. Stress 6, 113–126. doi: 10.1016/j.ynstr.2016.11.001, PMID: 28229114 PMC5314443

[ref4] Andersson R. Larsson L. Holmqvist K. Stridh M. Nyström M. (2017). One algorithm to rule them all? An evaluation and discussion of ten eye movement event-detection algorithms. Behav. Res. Methods 49, 616–637. doi: 10.3758/s13428-016-0738-9, PMID: 27193160

[ref5] Ataya A. F. Adams S. Mullings E. Cooper R. M. Attwood A. S. Munafò M. R. (2012). Internal reliability of measures of substance-related cognitive bias. Drug Alcohol Depend. 121, 148–151. doi: 10.1016/j.drugalcdep.2011.08.023, PMID: 21955365

[ref6] Baker T. B. Brandon T. H. Chassin L. (2004). Motivational influences on cigarette smoking. Annu. Rev. Psychol. 55, 463–491. doi: 10.1146/annurev.psych.55.090902.142054 14744223

[ref7] Berridge K. C. Robinson T. E. (2016). Liking, wanting, and the incentive-sensitization theory of addiction. Am. Psychol. 71, 670–679. doi: 10.1037/amp0000059, PMID: 27977239 PMC5171207

[ref8] Bonitz V. S. Gordon R. D. (2008). Attention to smoking-related and incongruous objects during scene viewing. Acta Psychol. 129, 255–263. doi: 10.1016/j.actpsy.2008.08.006, PMID: 18804752 PMC4058766

[ref9] Bradley B. Field M. Mogg K., & Houwer, J. de (2004). Attentional and evaluative biases for smoking cues in nicotine dependence: component processes of biases in visual orienting. Behav. Pharmacol., 15, 29–36. doi: 10.1097/00008877-200402000-00004, PMID: 15075624

[ref10] Brysbaert M. (2019). How many participants do we have to include in properly powered experiments? A tutorial of power analysis with reference tables. J. Cogn. 2:16. doi: 10.5334/joc.72, PMID: 31517234 PMC6640316

[ref11] Christou C. G. Aristidou P. (2017). Steering versus teleport locomotion for head mounted displays. In Paolis L. T. de Bourdot P. Mongelli A. (Eds.), Lecture notes in computer science. Augmented reality, virtual reality, and computer graphics (Vol. 10325, pp. 431–446). Cham: Springer International Publishing.

[ref12] Cohen J. (1988). Statistical power analysis for the behavioral sciences. 2nd edn. Hillsdale, NJ: Erlbaum.

[ref13] Cox W. M. Fadardi J. S. Pothos E. M. (2006). The addiction-stroop test: theoretical considerations and procedural recommendations. Psychol. Bull. 132, 443–476. doi: 10.1037/0033-2909.132.3.443, PMID: 16719569

[ref14] Cox L. S. Tiffany S. T. Christen A. G. (2001). Evaluation of the brief questionnaire of smoking urges (QSU-brief) in laboratory and clinical settings. Nicotine Tob. Res. 3, 7–16. doi: 10.1080/14622200020032051, PMID: 11260806

[ref15] Dar A. H. Wagner A. S. Hanke M. (2021). Remodnav: robust eye-movement classification for dynamic stimulation. Behav. Res. Methods 53, 399–414. doi: 10.3758/s13428-020-01428-x, PMID: 32710238 PMC7880959

[ref16] Dickerson S. S. Kemeny M. E. (2004). Acute stressors and cortisol responses: a theoretical integration and synthesis of laboratory research. Psychol. Bull. 130, 355–391. doi: 10.1037/0033-2909.130.3.355, PMID: 15122924

[ref17] Diemer J. Alpers G. W. Peperkorn H. M. Shiban Y. Mühlberger A. (2015). The impact of perception and presence on emotional reactions: a review of research in virtual reality. Front. Psychol. 6:26. doi: 10.3389/fpsyg.2015.00026, PMID: 25688218 PMC4311610

[ref18] Drobes D. J. Oliver J. A. Correa J. B. Evans D. E. (2019). “Attentional bias and smoking” in Neuroscience of nicotine. ed. Preedy V. R. (London: Academic Press), 145–150.

[ref19] Eastwood B. Bradley B. Mogg K. Tyler E. Field M. (2010). Investigating the effects of a craving induction procedure on cognitive bias in cannabis users. Addict. Res. Theory 18, 97–109. doi: 10.3109/16066350802699328

[ref20] Ebner-Priemer U. W. Trull T. J. (2009). Ecological momentary assessment of mood disorders and mood dysregulation. Psychol. Assess. 21, 463–475. doi: 10.1037/a0017075 19947781

[ref21] Fagerström K. (2012). Determinants of tobacco use and renaming the FTND to the Fagerström test for cigarette dependence. Nicotine Tobacco Res. 14, 75–78. doi: 10.1093/ntr/ntr137, PMID: 22025545

[ref22] Field M. Cox W. M. (2008). Attentional bias in addictive behaviors: a review of its development, causes, and consequences. Drug Alcohol Depend. 97, 1–20. doi: 10.1016/j.drugalcdep.2008.03.030, PMID: 18479844

[ref23] Field M. Marhe R. Franken I. H. A. (2014). The clinical relevance of attentional bias in substance use disorders. CNS Spectr. 19, 225–230. doi: 10.1017/S1092852913000321, PMID: 23663386

[ref24] Field M. Mogg K. Bradley B. P. (2004). Eye movements to smoking-related cues: effects of nicotine deprivation. Psychopharmacology 173, 116–123. doi: 10.1007/s00213-003-1689-2, PMID: 14663552

[ref25] Field M. Mogg K. Bradley B. P. (2005). Alcohol increases cognitive biases for smoking cues in smokers. Psychopharmacology 180, 63–72. doi: 10.1007/s00213-005-2251-1, PMID: 15834537

[ref26] Field M. Munafò M. R. Franken I. H. A. (2009). A meta-analytic investigation of the relationship between attentional bias and subjective craving in substance abuse. Psychol. Bull. 135, 589–607. doi: 10.1037/a0015843, PMID: 19586163 PMC2999821

[ref27] Field M. Rush M. Cole J. Goudie A. (2007). The smoking Stroop and delay discounting in smokers: effects of environmental smoking cues. J. Psychopharmacol. 21, 603–610. doi: 10.1177/0269881106070995, PMID: 17092980

[ref28] Field M. Werthmann J. Franken I. Hofmann W. Hogarth L. Roefs A. (2016). The role of attentional bias in obesity and addiction. Health Psychol. 35, 767–780. doi: 10.1037/hea0000405, PMID: 27505196

[ref29] Freeman T. P. Morgan C. J. A. Beesley T. Curran H. V. (2012). Drug cue induced overshadowing: selective disruption of natural reward processing by cigarette cues amongst abstinent but not satiated smokers. Psychol. Med. 42, 161–171. doi: 10.1017/S0033291711001139, PMID: 21733292

[ref30] Goodman W. K. Janson J. Wolf J. M. (2017). Meta-analytical assessment of the effects of protocol variations on cortisol responses to the Trier social stress test. Psychoneuroendocrinology 80, 26–35. doi: 10.1016/j.psyneuen.2017.02.030, PMID: 28292684

[ref31] Hajek P. Taylor T. McRobbie H. (2010). The effect of stopping smoking on perceived stress levels. Addiction 105, 1466–1471. doi: 10.1111/j.1360-0443.2010.02979.x 20528815

[ref32] Heatherton T. F. Kozlowski L. T. Frecker R. C. Fagerström K.-O. (1991). The Fagerström test for nicotine dependence: a revision of the Fagerstrom tolerance questionnaire. Br. J. Addict. 86, 1119–1127. doi: 10.1111/j.1360-0443.1991.tb01879.x, PMID: 1932883

[ref33] Het S. Rohleder N. Schoofs D. Kirschbaum C. Wolf O. T. (2009). Neuroendocrine and psychometric evaluation of a placebo version of the 'Trier social stress Test'. Psychoneuroendocrinology 34, 1075–1086. doi: 10.1016/j.psyneuen.2009.02.008, PMID: 19307062

[ref34] Hindocha C. Freeman T. P. Grabski M. Stroud J. B. Crudgington H. Davies A. C.. (2018). Cannabidiol reverses attentional bias to cigarette cues in a human experimental model of tobacco withdrawal. Addiction 113, 1696–1705. doi: 10.1111/add.14243, PMID: 29714034 PMC6099309

[ref35] Hogarth L. C. Mogg K. Bradley B. P. Duka T. Dickinson A. (2003). Attentional orienting towards smoking-related stimuli. Behav. Pharmacol. 14, 153–160. doi: 10.1097/01.fbp.0000063527.83818.9e, PMID: 12658076

[ref36] Huang Y. Liu Z. Ji H. Duan Z. Ling H. Chen J.. (2020). Attentional bias in methamphetamine users: a visual search task study. Addict. Res. Theory 28, 517–525. doi: 10.1080/16066359.2019.1708905

[ref37] JASP Team (2022). JASP (version 0.16.4)[computer software]. Available at: https://jasp-stats.org/ (Accessed March 17, 2023).

[ref38] Jeffreys H. (1961). Theory of probability. 3rd edn. Oxford: Oxford University Press.

[ref39] Kassel J. D. Stroud L. R. Paronis C. A. (2003). Smoking, stress, and negative affect: correlation, causation, and context across stages of smoking. Psychol. Bull. 129, 270–304. doi: 10.1037/0033-2909.129.2.270, PMID: 12696841

[ref40] Kim M. Lee T. H. Choi J.-S. Kwak Y. B. Hwang W. J. Kim T.. (2019). Dysfunctional attentional bias and inhibitory control during anti-saccade task in patients with internet gaming disorder: an eye tracking study. Prog. Neuro-Psychopharmacol. Biol. Psychiatry 95:109717. doi: 10.1016/j.pnpbp.2019.109717, PMID: 31351161

[ref41] Kim H. K. Park J. Choi Y. Choe M. (2018). Virtual reality sickness questionnaire (VRSQ): motion sickness measurement index in a virtual reality environment. Appl. Ergon. 69, 66–73. doi: 10.1016/j.apergo.2017.12.016, PMID: 29477332

[ref42] Kirschbaum C. Pirke K. M. Hellhammer D. H. (1993). The 'Trier social stress Test'--a tool for investigating psychobiological stress responses in a laboratory setting. Neuropsychobiology 28, 76–81. doi: 10.1159/000119004 8255414

[ref43] Knight H. C. Smith D. T. Knight D. C. Ellison A. (2016). Altering attentional control settings causes persistent biases of visual attention. Q. J. Exp. Psychol. 69, 129–149. doi: 10.1080/17470218.2015.1031144, PMID: 25801329

[ref44] Kotlyar M. Donahue C. Thuras P. Kushner M. G. O'Gorman N. Smith E. A.. (2008). Physiological response to a speech stressor presented in a virtual reality environment. Psychophysiology 45, 1034–1037. doi: 10.1111/j.1469-8986.2008.00690.x, PMID: 18778321

[ref45] Kudielka B. Hellhammer D. Kirschbaum C. (2007). “Ten years of research with the Trier social stress test—revisited” in Social neuroscience: Integrating biological and psychological explanations of social behavior. eds. Harmon-Jones E. Winkielman P. (New York: Guilford Press), 56–83.

[ref46] MacLeod C. Mathews A. Tata P. (1986). Attentional bias in emotional disorders. J. Abnorm. Psychol. 95, 15–20. doi: 10.1037/0021-843X.95.1.15 3700842

[ref47] Margraf J. Cwik J. C. (2017). Mini-DIPS Open Access: Diagnostisches Kurzinterview bei psychischen Störungen. Bochum: Forschungs- und Behandlungszentrum für psychische Gesundheit, Ruhr-Universität Bochum.

[ref48] McCarthy D. E. Gloria R. Curtin J. J. (2009). Attention bias in nicotine withdrawal and under stress. Psychol. Addict. Behav. 23, 77–90. doi: 10.1037/a0014288, PMID: 19290692 PMC2897737

[ref49] McEwen A. West R. McRobbie H. (2008). Motives for smoking and their correlates in clients attending stop smoking treatment services. Nicotine Tobacco Res. 10, 843–850. doi: 10.1080/14622200802027248, PMID: 18569758

[ref50] McKee S. A. Sinha R. Weinberger A. H. Sofuoglu M. Harrison E. L. R. Lavery M.. (2011). Stress decreases the ability to resist smoking and potentiates smoking intensity and reward. J. Psychopharmacol. 25, 490–502. doi: 10.1177/0269881110376694, PMID: 20817750 PMC3637660

[ref51] Mogg K. Bradley B. P. Field M., & Houwer, J. de (2003). Eye movements to smoking-related pictures in smokers: relationship between attentional biases and implicit and explicit measures of stimulus valence. Addiction, 98, 825–836. doi: 10.1046/j.1360-0443.2003.00392.x, PMID: . 12780371

[ref52] Narvaez Linares N. F. Charron V. Ouimet A. J. Labelle P. R. Plamondon H. (2020). A systematic review of the Trier social stress test methodology: issues in promoting study comparison and replicable research. Neurobiol. Stress 13:100235. doi: 10.1016/j.ynstr.2020.100235, PMID: 33344691 PMC7739033

[ref53] Nichter M. Nichter M. Carkoglu A. (2007). Reconsidering stress and smoking: a qualitative study among college students. Tob. Control. 16, 211–214. doi: 10.1136/tc.2007.019869, PMID: 17565143 PMC2598509

[ref54] Nummenmaa L. Hyönä J. Calvo M. G. (2006). Eye movement assessment of selective attentional capture by emotional pictures. Emotion 6, 257–268. doi: 10.1037/1528-3542.6.2.257, PMID: 16768558

[ref55] Nyström M. Holmqvist K. (2010). An adaptive algorithm for fixation, saccade, and glissade detection in eyetracking data. Behav. Res. Methods 42, 188–204. doi: 10.3758/BRM.42.1.188, PMID: 20160299

[ref56] Pekal J. Laier C. Snagowski J. Stark R. Brand M. (2018). Tendencies toward internet-pornography-use disorder: differences in men and women regarding attentional biases to pornographic stimuli. J. Behav. Addict. 7, 574–583. doi: 10.1556/2006.7.2018.70, PMID: 30203692 PMC6426393

[ref57] Robinson T. E. Berridge K. C. (1993). The neural basis of drug craving: an incentive-sensitization theory of addiction. Brain Res. Rev. 18, 247–291. doi: 10.1016/0165-0173(93)90013-P, PMID: 8401595

[ref58] Rooke S. E. Hine D. W. Thorsteinsson E. B. (2008). Implicit cognition and substance use: a meta-analysis. Addict. Behav. 33, 1314–1328. doi: 10.1016/j.addbeh.2008.06.009, PMID: 18640788

[ref59] Ruisoto P. Contador I. (2019). The role of stress in drug addiction. An integrative review. Physiol. Behav. 202, 62–68. doi: 10.1016/j.physbeh.2019.01.022, PMID: 30711532

[ref60] Schröder B. Mühlberger A. (2022). Assessing the attentional bias of smokers in a virtual reality anti-saccade task using eye tracking. Biol. Psychol. 172:108381. doi: 10.1016/j.biopsycho.2022.108381, PMID: 35710075

[ref61] Schubert T. Friedmann F. Regenbrecht H. (2001). The experience of presence: factor analytic insights. Presence 10, 266–281. doi: 10.1162/105474601300343603

[ref62] Schultz M. E. Fronk G. E. Jaume N. Magruder K. P. Curtin J. J. (2022). Stressor-elicited smoking and craving during a smoking cessation attempt. J. Psychopathol. Clin. Sci. 131, 73–85. doi: 10.1037/abn0000702, PMID: 34881919 PMC8979188

[ref63] SensoMotoric Instruments (2016). Eye tracking HMD based on HTC Vive - technical specification. Teltow, Germany: SensoMotoric Instruments GmbH.

[ref64] Shiban Y. Diemer J. Brandl S. Zack R. Mühlberger A. Wüst S. (2016). Trier social stress test in vivo and in virtual reality: dissociation of response domains. Int. Psychophysiol. 110, 47–55. doi: 10.1016/j.ijpsycho.2016.10.008, PMID: 27742258

[ref65] Sinha R. (2001). How does stress increase risk of drug abuse and relapse? Psychopharmacology 158, 343–359. doi: 10.1007/s002130100917 11797055

[ref66] Sinha R. (2007). The role of stress in addiction relapse. Curr. Psychiatry Rep. 9, 388–395. doi: 10.1007/s11920-007-0050-6 17915078

[ref67] Taylor G. M. J. Sawyer K. Kessler D. Munafò M. R. Aveyard P. Shaw A. (2021). Views about integrating smoking cessation treatment within psychological services for patients with common mental illness: a multi-perspective qualitative study. Health Exp. 24, 411–420. doi: 10.1111/hex.13182, PMID: 33368996 PMC8077097

[ref68] Twyman L. Bonevski B. Paul C. Bryant J. (2014). Perceived barriers to smoking cessation in selected vulnerable groups: a systematic review of the qualitative and quantitative literature. BMJ Open 4:e006414. doi: 10.1136/bmjopen-2014-006414, PMID: 25534212 PMC4275698

[ref69] van Bockstaele B. Verschuere B. Tibboel H. Houwer J. De Crombez G. Koster E. H. W. (2014). A review of current evidence for the causal impact of attentional bias on fear and anxiety. Psychol. Bull., 140, 682–721. doi: 10.1037/a0034834, PMID: 24188418

[ref70] Vollstädt-Klein S. Loeber S. Winter S. Leménager T. Goltz C. Von Der Dinter C.. (2011). Attention shift towards smoking cues relates to severity of dependence, smoking behavior and breath carbon monoxide. Eur. Addict. Res., 17, 217–224. doi: 10.1159/000327775, PMID: 21606649

[ref71] Wagenmakers E.-J. Marsman M. Jamil T. Ly A. Verhagen J. Love J.. (2018). Bayesian inference for psychology. Part I: theoretical advantages and practical ramifications. Psychon. Bull. Rev. 25, 35–57. doi: 10.3758/s13423-017-1343-3, PMID: 28779455 PMC5862936

[ref72] Wertz J. M. Sayette M. A. (2001). Effects of smoking opportunity on attentional bias in smokers. Psychol. Addict. Behav. 15, 268–271. doi: 10.1037/0893-164X.15.3.268, PMID: 11563808 PMC2632777

[ref73] Wiemers U. S. Schoofs D. Wolf O. T. (2013). A friendly version of the trier social stress test does not activate the HPA axis in healthy men and women. Stress 16, 254–260. doi: 10.3109/10253890.2012.714427 22813431

[ref74] Yan X. Jiang Y. Wang J. Deng Y. He S. Weng X. (2009). Preconscious attentional bias in cigarette smokers: a probe into awareness modulation on attentional bias. Addict. Biol. 14, 478–488. doi: 10.1111/j.1369-1600.2009.00172.x, PMID: 19740368

[ref75] Yaxley R. H. Zwaan R. A. (2005). Attentional bias affects change detection. Psychon. Bull. Rev. 12, 1106–1111. doi: 10.3758/bf03206451 16615336

[ref76] Zhang M. Ying J. Wing T. Song G. Fung D. S. S. Smith H. (2018). A systematic review of attention biases in opioid, cannabis, stimulant use disorders. Int. J. Environ. Res. Public Health 15:1138. doi: 10.3390/ijerph15061138, PMID: 29857586 PMC6025086

[ref001] Zimmer P. Buttlar B. Halbeisen G. Walther E. Domes G. (2019). Virtually stressed? A refined virtual reality adaptation of the Trier Social Stress Test (TSST) induces robust endocrine responses. Psychoneuroendocrinology 101, 186–192. doi: 10.1016/j.psyneuen.2018.11.010 30469086

